# Detection rate of contrast-enhanced brain magnetic resonance imaging in patients with cognitive impairment

**DOI:** 10.1371/journal.pone.0289638

**Published:** 2023-08-07

**Authors:** Leehi Joo, Chong Hyun Suh, Woo Hyun Shim, Seon-Ok Kim, Jae-Sung Lim, Jae-Hong Lee, Ho Sung Kim, Sang Joon Kim

**Affiliations:** 1 Department of Radiology, Korea University Guro Hospital, Seoul, Republic of Korea; 2 Department of Radiology and Research Institute of Radiology, Asan Medical Center, University of Ulsan College of Medicine, Seoul, Republic of Korea; 3 Department of Clinical Epidemiology and Biostatistics, Asan Medical Center, University of Ulsan College of Medicine, Seoul, Republic of Korea; 4 Department of Neurology, Asan Medical Center, University of Ulsan College of Medicine, Seoul, Republic of Korea; Kaohsuing Medical University Hospital, TAIWAN

## Abstract

**Introduction:**

The number of brain MRI with contrast media performed in patients with cognitive impairment has increased without universal agreement. We aimed to evaluate the detection rate of contrast-enhanced brain MRI in patients with cognitive impairment.

**Materials and methods:**

This single-institution, retrospective study included 4,838 patients who attended outpatient clinics for cognitive impairment evaluation and underwent brain MRI with or without contrast enhancement from December 2015 to February 2020. Patients who tested positive for cognitive impairment were followed-up to confirm whether the result was true-positive and provide follow-up management. Detection rate was defined as the proportion of patients with true-positive results and was compared between groups with and without contrast enhancement. Individual matching in a 1:2 ratio according to age, sex, and year of test was performed.

**Results:**

The overall detection rates of brain MRI with and without contrast media were 4.7% (57/1,203; 95% CI: 3.6%–6.1%) and 1.8% (65/3,635; 95% CI: 1.4%–2.3%), respectively (P<0.001); individual matching demonstrated similar results (4.7% and 1.9%). Among 1,203 patients with contrast media, 3.6% was only detectable with the aid of contrast media. The proportion of patients who underwent follow-up imaging or treatment for the detected lesions were significantly higher in the group with contrast media (2.0% and 0.6%, P < .001).

**Conclusions:**

Detection rate of brain MRI for lesions only detectable with contrast media in patients with cognitive impairment was not high enough and further study is needed to identify whom would truly benefit with contrast media.

## Introduction

Dementia is a disorder characterized by a cognitive decline [1] and this global concern is continuously increasing with the aging of the population. [2, 3] Although guidelines may vary, structural neuroimaging is not always recommended to solely diagnose dementia. However, the American Academy of Neurology [4] recommends structural neuroimaging (noncontrast CT or MRI) in the initial evaluation of patients with suspected dementia as it may assist in the diagnosis, identifying other possible causes of cognitive decline such as brain neoplasm or subdural hematoma. The European Federation of the Neurological Societies guidelines [5, 6] also recommend that structural imaging should be performed at least once in every patient with cognitive impairment. The primary purposes of structural imaging are to exclude potentially treatable or reversible causes of dementia, identify vascular lesions, and evaluate specific patterns of different types of dementia [6].

However, the tendency appears to shift toward assessing specific types of dementia rather than just to exclude other significant intracranial abnormalities using brain MRI [5, 7–11]. The minimum set regarded as essential sequences in an MRI procedure include three-dimensional (3D) T1-weighted gradient echo; turbo/fast spin echo T2-weighted, fluid-attenuated inversion recovery (FLAIR); and T2*-gradient echo [6, 9]. Although a contrast enhancement study may be better to evaluate neoplasms, infectious or inflammatory diseases, noncontrast study is usually considered sufficient for evaluating patients with suspected dementia [12]. That is, the routine use of contrast media is not indicated in initial imaging assessment of dementia according to current guidelines [6, 12].

However, the demand and actual number of contrast enhancement studies have been increasing based on the preference of physicians (S1 Fig) and there were no studies evaluating the detection rate of contrast-enhanced brain MRI in a large cohort. Therefore, this study aimed to evaluate the usefulness of contrast enhancement study in patients with cognitive impairment by calculating the detection rates.

## Materials and methods

### Study population

**Fig 1:**
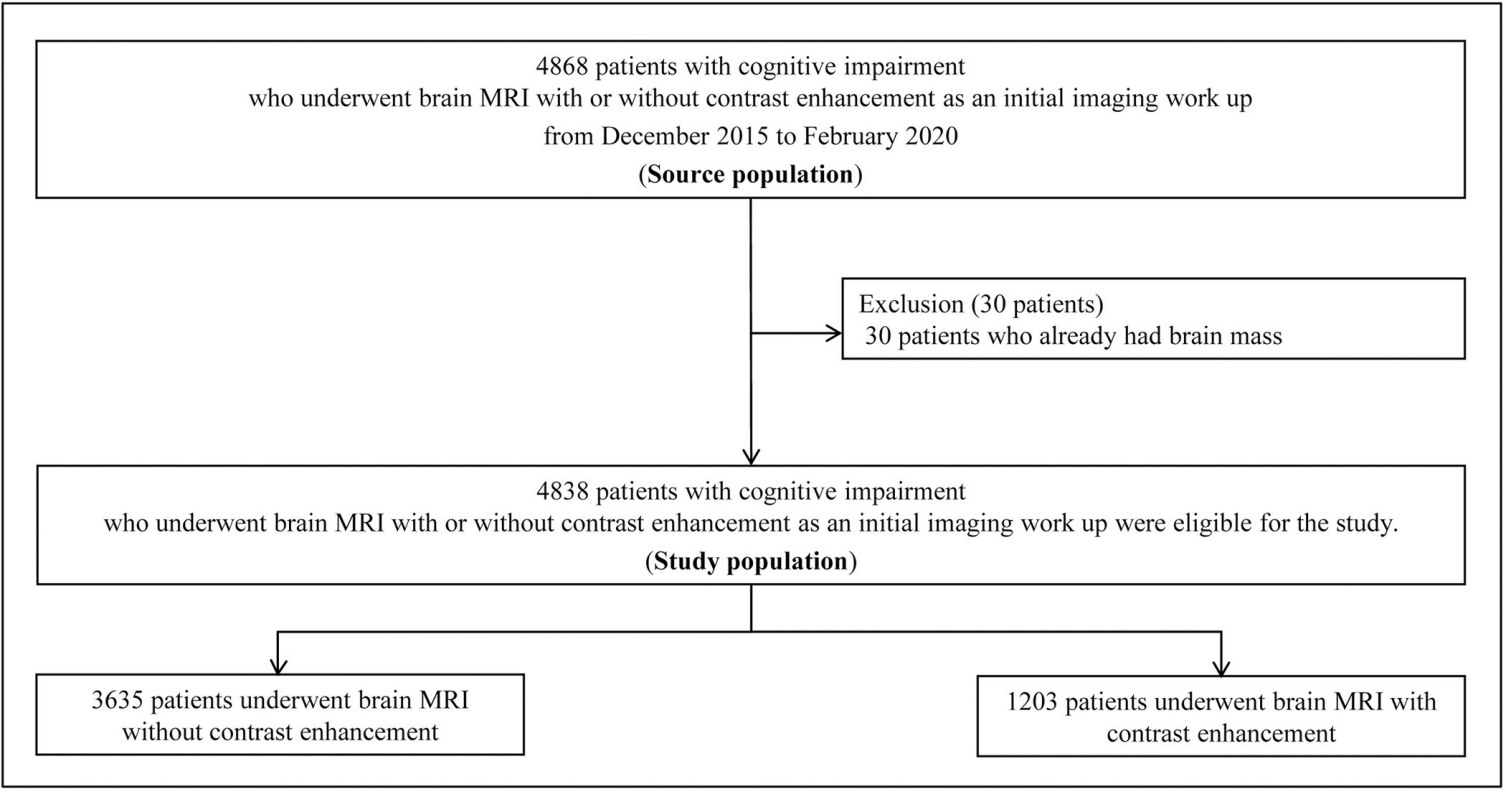
Flow diagram of the study population.

This single-institution and observational study was approved by our institutional review board. The requirement for a written informed consent was waived due to the retrospective study design. Our hospital’s electronic medical records were searched to identify patients who attended outpatient clinics for cognitive impairment evaluation and underwent brain MRI with or without contrast media from December 2015 to February 2020. Patients with cognitive impairment who attended outpatient clinics for the first time and underwent brain MRI with or without contrast media as an imaging workup within one month of attendance were included in the study. Contrast enhancement was solely determined according to referral physicians’ opinion and preference. Patients who already developed brain masses were excluded. Fig 1 presents the flow diagram of the study population.

### Brain MRI including contrast-enhanced images

MRI was performed with a 3.0-T system (Achieva or Ingenia; Philips Medical Systems, Best, The Netherlands) using an eight-channel sensitivity-encoding head coil. The sequences for examining patients with cognitive impairment were as follows: 3D T1-weighted image, FLAIR, T2-weighted image, susceptibility-weighted image, and diffusion-weighted image. Inclusion of contrast-enhanced 3D T1-weighted images was determined based on the referral physician’s preference. When contrast-enhanced 3D T1-weighted images was included, gadobutrol (Gadovist; Bayer- Schering Pharma, Berlin, Germany) 0.01 mmol/kg was intravenously injected at a rate of 1.5 mL/sec. A contrast-enhanced 3D T1 images were obtained with following parameters: TR/TE, 9.9/4.6 ms; flip angle 8; field of view, 220 x 220; matrix size, 256 x 256; and 3 mm slice thickness without gap. A contrast-enhanced 3D T1-weighted images had 3-mm slice thickness on the picture archiving and communication system; images with 1-mm slice thickness were accessible using a server-based workstation (AQNet; TeraRecon, Durham, NC).

### Brain MRI analysis and reference standards

Reports of brain MRI for imaging evaluation of patients with cognitive impairment were reviewed by an experienced neuroradiologist (XXX with 9 years of experience in neuroradiology) with expertise in dementia. Reports with positive results on contrast-enhanced images were reviewed, and the proportion of positive brain MRI results was calculated. For brain MRI with contrast media, decision of positive results (test positive) was solely based on whether a lesion shows contrast enhancement or not. Lesions that showed contrast enhancement including tumorous condition such as meningioma, metastasis, glioma, pituitary adenoma, lymphoma, neurogenic tumor, and bone tumor, and inflammation such as encephalitis, meningitis, and vascular malformation were regarded as positive test results. Other reported findings that were irrelevant to contrast enhancement such as brain atrophy, non-specific white matter lesions, microbleeds and old infarction/hemorrhage were not considered as positive test results. Reports without positive results defined on contrast-enhanced images were considered as negative test results. For brain MRI without contrast media, a positive test result was determined if the previously defined positive lesions on contrast-enhanced images were present. A negative test result was determined if the positive lesions were not present on the reports.

All test positive images of brain MRI with or without contrast media were reviewed by two neuroradiologists (XXX and XXX with 5 and 9 years of experience in neuroradiology, respectively) who were blinded to other relevant information including the clinical and imaging aspects. When discrepancies were noted, the images were reviewed by the most experienced neuroradiologist (XXX with 35 years of experience in neuroradiology). Whether the positive lesions are detectable without contrast-enhanced images and whether the location of the lesions may anatomically cause cognitive impairment were also evaluated. We assumed the lesions might causative if they are located at medial temporal lobe, frontal lobe, or fornix with mass effects.

Patients who tested positive were followed-up to identify their treatment course. Brain tumors were considered to be present (true-positive result) when the clinical and radiologic data were in consensus or the lesion was confirmed by operation or biopsy. Other positive results such as inflammation or vascular malformation were considered to be present (true-positive result) when the clinical-radiologic data were in consensus. For patients with brain tumors, the detailed information including patients’ age, sex, and size and location of tumors were documented.

### Outcomes

The primary outcome was detection rate of contrast-enhanced brain MRI as a part of imaging evaluation in patients with cognitive impairment. Detection rate was defined as the proportion of patients with true-positive results among all patients (number of true-positive results divided by the total number of patients) [13]. The proportions of true-positive results were compared between two groups of patients who underwent brain MRI with and without contrast media. Additionally, detection rates for the lesions that are only detectable on contrast-enhanced images and for the lesions that might cause cognitive impairment were also evaluated. The secondary outcome was false referral rate, defined as the proportion of patients with false-positive results among all patients (number of false-positive results divided by the total number of patients). Moreover, the proportion of patients underwent follow-up imaging or treatment was evaluated. Hypersensitivity reaction was classified into mild, moderate, and severe categories by using American College of Radiology criteria [14]. Meanwhile, this study carried out a patient level analysis.

### Statistical analysis

As the age, sex, and the year of examination of the two study groups may differ, individual matching was used to reduce the potential selection bias. Subgroup analysis according to the mean age of the study population and sex was done (S1 Table). Patients with contrast media and those without contrast media were matched in a 1:2 ratio according to age (±5 years), sex, and year of test (±2 years). Matching was performed using the greedy algorithm. The balance of covariates obtained from matching was assessed by evaluating the standardized differences. Logistic regression was used to compare the detection rate according to the need for a contrast media. The generalized estimating equation method was used for all analyses to account for exchangeable correlation between lesions within the same patient before matching and lesions within matched pairs after matching. The correlation structure type was determined as the structure with the smallest quasilikelihood information criterion (QIC) among Unstructured, Exchangeable and Independent type [15]. Mann Whitney U test was additionally used in subgroup analysis for comparing the size of meningiomas. SAS software, version 9.4 (SAS Institute), was used to perform all analyses.

## Results

### Patients’ characteristics

Over 4,838 patients attended the outpatient clinic for cognitive impairment evaluation from December 2015 to February 2020, and 5,297 examinations were undergone as imaging workups. Thirty patients who already had known brain mass was excluded. As most of the patients had only one examination (4,609/4,838, 95%) 5,267 examinations of 4,838 patients with their initial examinations were included in the final analysis (Fig 1 and Table 1). Among them, 1203 patients underwent brain MRI with contrast media.

**Table 1:** Baseline characteristics.

	Total (n = 4,838)			Individual matching (n = 3,523)		
	With enhancement (n = 1,231) (%)	Without enhancement (n = 3,635) (%)	Standardized difference	With enhancement (n = 1,203) (%)	Without enhancement (n = 2,320) (%)	Standardized difference
Age	67.5±11.8	70.9±10.4	0.3000	67.5±11.8	68.4±11.1	0.0790
Sex						
Female	688 (57.2)	2,310 (63.6)	0.1300	688 (57.2)	1,323 (57.2)	0.0030
Male	515 (42.8)	1,325 (36.4)		515 (42.8)	997 (43.0)	
Year						
2015	17 (1.4)	84 (2.3)	0.1820	17 (1.4)	32 (1.4)	0.0970
2016	237 (19.7)	881 (24.2)		237 (19.7)	466 (20.1)	
2017	254 (21.1)	824 (22.7)		254 (21.1)	534 (23.0)	
2018	280 (23.3)	855 (23.5)		280 (23.3)	569 (24.5)	
2019	370 (30.8)	901 (24.8)		370 (30.8)	662 (28.5)	
2020	45 (3.7)	90 (2.5)		45 (3.7)	57 (2.5)	

### Detection rate of brain MRI with contrast media

Table 2 presents the overall detection rate and detection rates according to each positive result. Fifty-seven of 1,203 examinations with contrast media were considered as true-positive; the overall detection rate of contrast-enhanced brain MRI was 4.7% (57/1,203; 95% CI: 3.6%–6.1%). True positive results included meningioma (24/1,203, 2.0%), metastasis (6/1,203, 0.5%), glioma (1/1,203, 0.08%), pituitary adenoma (2/1,203, 0.17%), lymphoma (2/1,203, 0.17%), neurogenic tumor (1/1,203, 0.08%), and others (21/1,203, 1.746%) such as bone tumor, encephalitis, vascular malformation, post-ictal change, or epidermoid cyst. The false referral rate of contrast-enhanced brain MRI was 0.17% (2/1,203, 95% CI: 0.02%–0.6%).

**Table 2:** Detection rate of MRI with or without contrast enhancement in patients with cognitive impairment.

	Total (n = 4,838)			Individual matching (n = 3,523)		
	With enhancement (n = 1,203) (%)	Without enhancement (n = 3,635) (%)	*P* value	With enhancement (n = 1,203) (%)	Without enhancement (n = 2,320) (%)	*P* value
Overall detection rate	57 (4.7) [95% CI: 3.6–6.1]	65 (1.8) [95% CI: 1.4–2.3]	< .001	57 (4.7) [95% CI: 3.6–6.1]	45 (1.9) [95% CI: 1.4–2.6]	< .001
Subgroup (detection rate)						
Meningioma	24 (2.0)	32 (0.88)	.002	24 (2.0)	23 (0.99)	.022
Metastasis	6 (0.50)	2 (0.06)	.004	6 (0.50)	2 (0.09)	.052
Glioma	1 (0.08)	5 (0.14)	1.000	1 (0.08)	2 (0.09)	.976
Pituitary adenoma	2 (0.17)	3 (0.08)	.604	2 (0.17)	2 (0.09)	.546
Lymphoma	2 (0.17)	2 (0.06)	.259	2 (0.17)	1 (0.04)	.325
Neurogenic tumor	1 (0.08)	4 (0.11)	1.000	1 (0.08)	3 (0.13)	.679
Others^a^	21 (1.8)	17 (0.45)	< .001	21 (1.8)	12 (0.52)	< .003
Treatment or follow-up imaging	24 (2.0)	22 (0.61)	< .001	24 (2.0)	14 (0.60)	.001

Note: Logistic regression with generalized estimating equations method was used to compare the detection rate according to need for contrast enhancement.

CI = confidence interval; MRI = magnetic resonance imaging

^a^ Others: bone tumor, encephalitis, meningitis, vascular malformation, post-ictal change, epidermoid cyst

Among 1,203 examinations with contrast media, 24 showing meningiomas (24/1,203, 2.0%; median 13 mm, range 5–68 mm) included 27 lesions: 22 solitary lesions, 1 case with 2 lesions, and 1 case with 3 lesions. Of the 27 meningiomas, 23 lesions were located in the supratentorial fossa, 3 were in the posterior fossa, and 1 was found in the tentorium cerebelli. The most common location of meningiomas was the convexity (n = 15). The other common tumor locations were as follows: falx (n = 3), petrous ridge (n = 2), sphenoid ridge (n = 3), tuberculum sellae (n = 2), and right tentorium cerebelli (n = 1). Among 24 exams with meningiomas, 14 (14/24, 58%) were detectable only on contrast-enhanced images. Ten meningiomas that were detectable without contrast-enhanced images were significantly larger in size (median, 19 mm vs. 10 mm; interquartile range, 15–30 vs. 8–13 mm; *P* = .026). The proportion of convexity meningiomas was higher in the group that had detectable lesions only on contrast-enhanced images but had no significant difference (64% vs. 50%, *P* = .493). Otherwise, there was no suspicious predilection in location with respect to detectability on images with or without contrast media.

**Fig 2:**
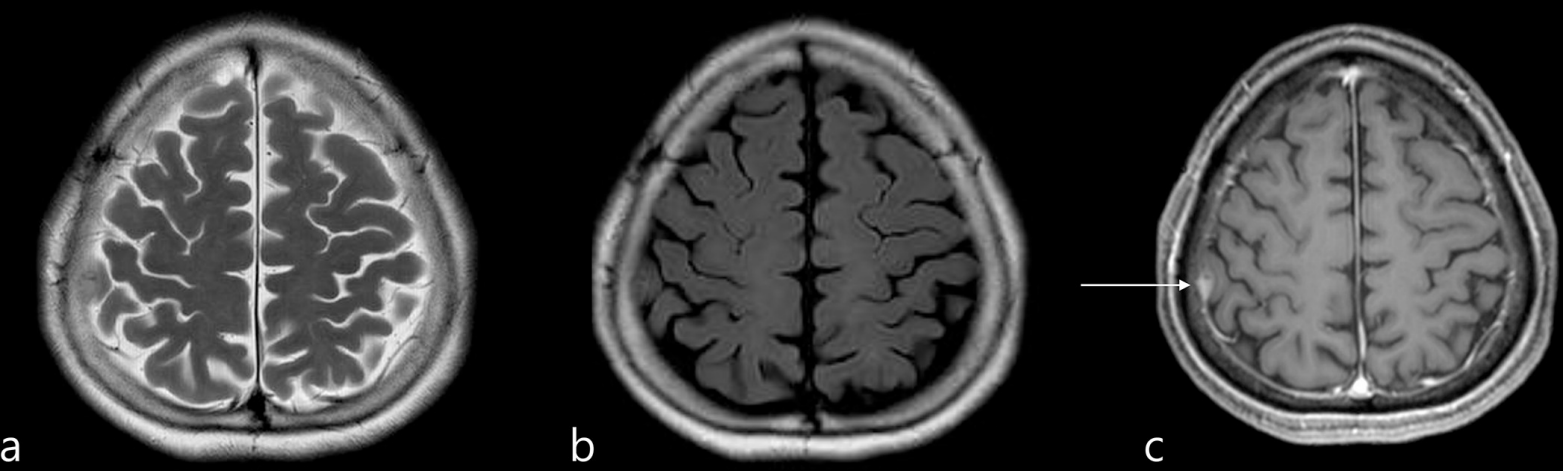
(a, b, c) Images in a 66-year-old female patient with a small extra-axial mass at the right parietal convexity suggestive of meningioma (arrow), which became noticeable with the aid of contrast enhancement study. (a, T2 weighted image; b, FLAIR; c, contrast-enhanced T1 weighted image).

**Fig 3:**
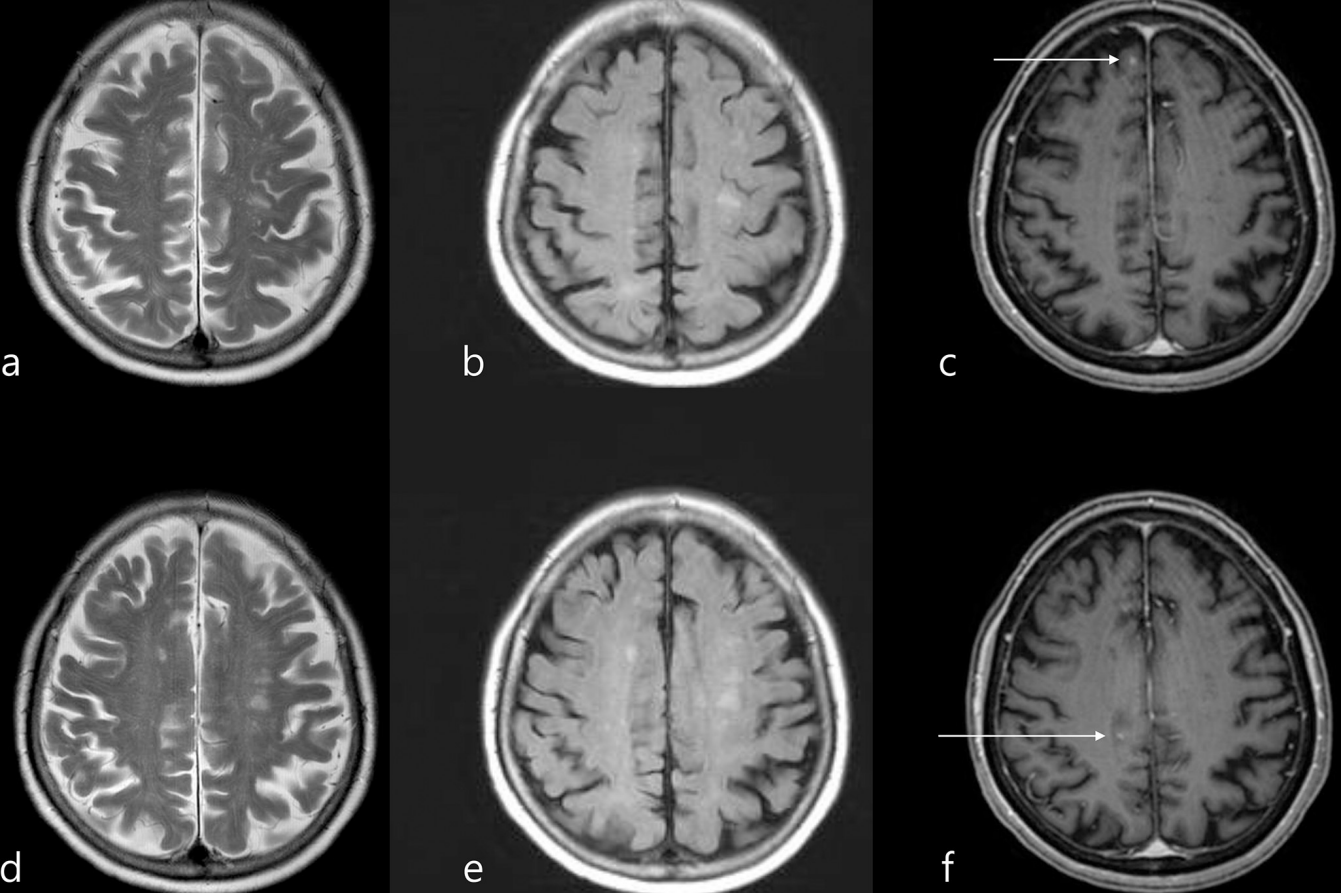
Images in a 75-year-old female patient with previous histories of right breast cancer and right renal cell carcinoma (clear cell type) with systemic metastases (retroperitoneum, lymph node, and lung). Contrast-enhanced T1 weighted images showed multiple tiny nodular enhancing lesions suggestive of brain metastases (one of them on (c)), which were not detectable on axial T2 weighted images (a) and FLAIR images (b).

**Fig 4:**
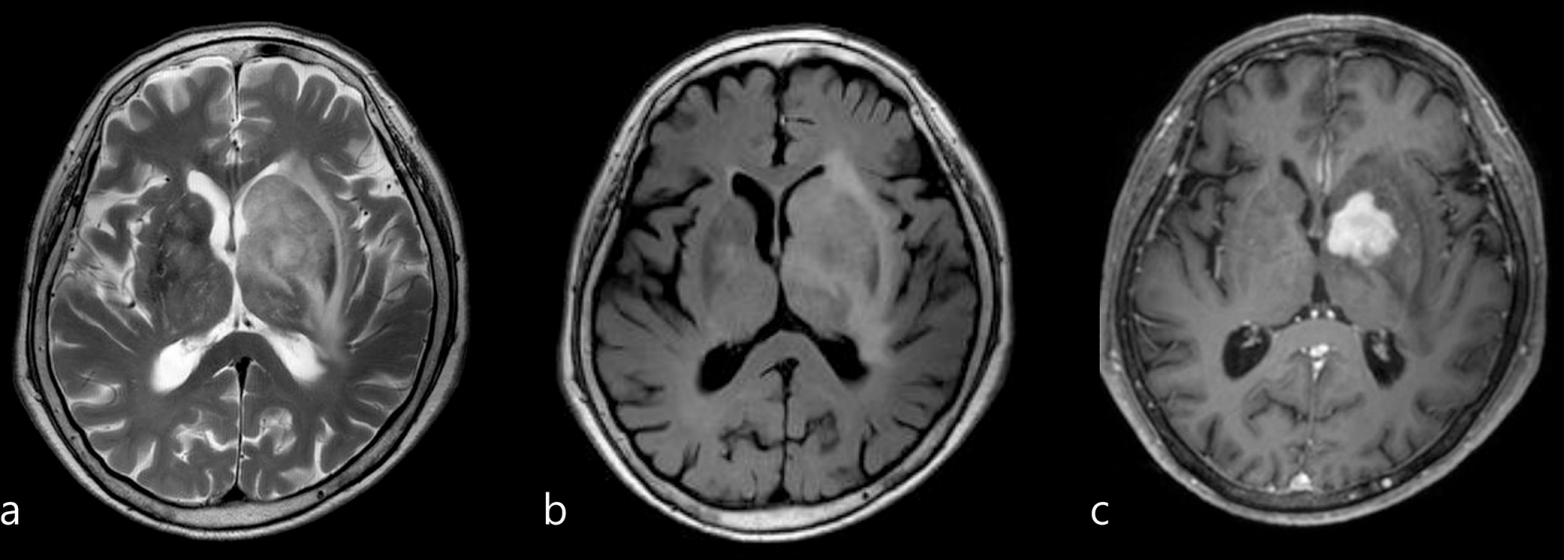
Images in an 86-year-old female patient presented with rapidly progressive dementia. Infiltrative high signal intensity lesion was noted on axial T2 weighted image (a) and FLAIR (b) with mass effect and edema involving left basal ganglia. Homogeneous enhancing mass was noted at the left basal ganglia on axial contrast-enhanced T1 weighted images (c). Lymphoma was suggested with the aid of contrast enhancement study rather than high grade glioma.

Among 1,203 examinations with contrast media, 6 showed metastases (6/1,203, 0.5%) in heterogeneous locations and varying sizes: 4 cases in the left frontal lobe (63 mm), left basal ganglia (41 mm), left parietal lobe (21 mm), and right parietal lobe (5 mm), respectively; 1 case with multiple lesions in the right frontal, parietal, and bilateral cerebellar hemispheres (largest diameter: 3 mm); and 1 case with diffuse leptomeningeal seeding. Among 6 with metastases, 3 (3/6, 50%) has prior history of cancer including lung cancer, breast cancer, and renal cell carcinoma. Four (4/6, 67%) had metastases detectable only on contrast-enhanced images, while 2 (2/6, 33%) with large masses (41 mm, 63 mm) developed metastases that were detectable without contrast-enhanced images. Representative cases are illustrated in Figs 2–4. Among 57 positive findings in patients with contrast media, 24.6% (14/57) was detectable without contrast media and 75.4% (43/57) was only detectable on contrast enhanced images.

Among 1,203 patients with contrast-enhanced images, 14 patients (14/1,203, 1.2%) had lesions that were already detectable without contrast media. Out of them, 2 patients had lesions located where they might cause cognitive impairment. Therefore, it is estimated that there was 0.2% (2/1,203) of patients who need not have undergone contrast enhancement. Among 1,203 patients with contrast-enhanced images, 43 patients (43/1,203, 3.6%) had lesions that were only detectable with the aid of contrast media. Out of them, 14 patients had lesions where they might cause cognitive impairment. That is, it is estimated that 1.2% (14/1,203) of patients would have missed lesions without contrast media. Additionally, 8 of 14 patients were presented with rapidly progressive cognitive impairment or had additional atypical symptoms including abnormal behavior or seizure like motion with or without previous cancer history and 9 of 14 patients were aged over 70. Taken together, thirteen patients out of 14 patients had one of the two factors (atypical presentation with or without previous cancer history and old age).

### Safety of brain MRI with contrast media

Among the 1,203 contrast-enhanced MR examinations, hypersensitivity reaction was reported in 5 patients, with an overall prevalence of 0.42% (5/1,203). In terms of severity, 4 patients were classified as mild, with an overall prevalence of 0.33% (4/1,203) and 1 patient were classified as moderate, with an overall prevalence of 0.08% (1/1,203).

### Detection rate of brain MRI without contrast media

Over 65 of 3,635 examinations without contrast media were true-positive (Table 2). The overall detection rate of brain MRI without contrast media was 1.8% (95% CI: 1.4%–2.3%). True positive results included meningioma (32/3,635, 0.88%), metastasis (2/3,635, 0.06%), glioma (5/3,635, 0.14%), pituitary adenoma (3/3,635, 0.08%), lymphoma (2/3,635, 0.06%), neurogenic tumor (4/3,635, 0.11%), and others (17/3,871, 0.45%). The false referral rate of brain MRI without contrast media was 0.028% (1/3,635, 95% CI: 0.00%–0.15%). This was not statistically different from that of with contrast media (P = .094). Among the all patients with non-contrast-enhanced images (n = 4,838), 79 patients (79/4,838, 1.6%) had positive findings that were detectable without contrast enhancement.

### Individual matching and comparison of detection rate of brain MRI with or without contrast media

After matching the patients with and without contrast media in a 1:2 ratio according to age, sex, and year of examination, we compared the detection rates in two groups: 1,203 examinations with contrast media and 2,320 examinations without contrast media. Their overall detection rates were 4.7% (57/1,203, [95% CI: 3.6%–6.1%]) and 1.9% (45/2,320, [95% CI: 1.4%–2.6%]), respectively, which was significantly higher in the group with contrast media (*P* < .001). The subgroup detection rate for meningiomas was significantly higher in the group that underwent brain MRI with contrast media (24/1,203, 2%, P = 0.025), while that for metastases which was also higher in the group that underwent brain MRI with contrast media showed borderline significance (6/1,203, 0.5%, *P* = .052).

### The proportion of patients underwent follow-up imaging or treatment

Among 4,838 patients, 24 (2%) and 22 (0.61%) patients who had brain MRI with and without media underwent follow-up imaging or treatment, including 19 (1.6%) and 11 (0.3%) patients who underwent treatment. Among 3,523 patients with individual matching, those who underwent a contrast enhancement study showed significantly higher rates of undergoing follow-up imaging or treatment than those without contrast enhancement study: 2.0% (24/1,203) and 0.6% (14/2,320) of patients underwent follow-up imaging or treatment, respectively (*P* < .001), while 1.6% (19/1,203) and 0.3% (8/2,320) patients underwent treatment, respectively (*P* = .001).

## Discussion

Increasing numbers of contrast enhancement studies have been performed for imaging evaluation of dementia without a widely established agreement. We evaluated the detection rates of brain MRI with or without contrast media in 4,838 consecutive patients who attended outpatient clinics for cognitive impairment evaluation. The overall detection rates of brain MRI with and without contrast media were 4.7% and 1.8%, respectively, (*P* < .001); individual matching demonstrated similar results (4.7% and 1.9%, respectively). Among the patients with contrast-enhanced images (n = 1,203), 43 patients (3.6%) had lesions that were only detectable with the aid of contrast media. However, the majority of these were meningiomas that were likely asymptomatic. Therefore, detection rate of brain MRI for lesions only detectable with contrast media in patients with cognitive impairment was not high enough and it is relatively reasonable not to use contrast media in patients with cognitive impairment. Further study is needed to develop a strategy for identifying patients whom would truly benefit from contrast media considering factors such as atypical presentation, previously known malignancy or older age.

True-positive findings used in the definition of detection rates may refer to incidental findings in the patients who underwent brain MRI for cognitive impairment evaluation. The detection rates we presented (4.7% with contrast media) were within the reported rates of incidental findings on brain MRI (1.4%–22%) [16–19]. The overall and the two most common subgroup (meningioma and metastasis) detection rates were significantly higher with contrast media. Meningioma comprised the largest portion of the overall detection rates (2.0% with contrast media), which was in line with a well-known fact that meningioma is the most common primary central nervous system tumors [20]. Incidental meningioma is one of common findings: 2.5% by [21] and 0.6% [19]. No significant difference was observed in the median sizes of meningiomas between the groups with and without contrast media. The mean size of meningiomas that were detectable only with the aid of contrast media was significantly smaller compared with the other (*P* = .026). Metastasis was the second most common subgroup of positive results. Metastases could not have been common positive findings in the previous studies that investigated incidental findings on brain MRI without contrast media [16, 22–24].

Detection rates can be finally meaningful when a detected lesion has potential risk of affecting patient’s prognosis, thus increasing the need to perform a follow-up imaging or treatment. Bos et al. [21] reported that 3.2% of the participants were referred to medical specialists due to the incidental detection of lesions and mostly followed the wait-and-see policy or were discharged after a single hospital visit (76.6%, 144/188; 95% CI: 70.1%–82.1%). Meanwhile, 2.0% of patients with contrast-enhanced images underwent follow-up imaging (0.4%) or treatment (1.6%) in our study. Whether there was improvement in cognitive impairment after treatment was no longer evaluated in our study. Moreover, the prevalence of lesions that underwent follow-up imaging or treatment was significantly higher with contrast enhancement study in our study (2.0% with enhancement vs. 0.6% without enhancement with individual matching, *P* = .001). Incidental detection of a smaller lesion is rather critical when the lesion is metastatic considering the optimal time for treatment and patient prognosis. However, it is insufficient to prove the necessity of contrast media in patients with cognitive impairment as it may just lead to overdiagnosis and unnecessary treatments [17, 25, 26]. According to the result from our age-based subgroup analysis (S2 Table), statistical significance was newly observed with contrast media in the older age group (>70 years) regarding detection rate of metastasis as well as proportion of treatment or follow-up imaging, which was not observed in the other age group (<70 years). Though contrast media may be more helpful in older age group considering the higher detection rate of incidental metastasis and higher proportion of treatment or follow-up imaging with contrast media, further study is needed to establish a justifiable indication of contrast media.

MRI with contrast media may raise clinical issues such as the adverse effects of gadolinium-based contrast media (GBCM), from mostly mild physiologic reactions to rare but severe life-threatening situations, including warmth, pain, allergic-like reaction, anaphylactoid reaction, and potential harm in the renal function [27]. Allergic-like reactions associated with the use of GBCM are not common (0.004%–0.7%) [27], and severe life-threatening anaphylactic reactions are rarer (0.001%–0.01%) [14, 28, 29]. In our study, an overall prevalence was 0.41% (5/1,231), which was similar with previous studies [30]. Although the association of GBCM and nephrogenic systemic fibrosis is generally accepted, GBCM at dosages approved for MRI are not regarded as nephrotoxic [27]. In spite of the issue concerning deposition of GBCM, approved macrocyclic agents that we used are known as safer ones because they exhibit lower levels of deposition than approved acyclic agents [31]. Contrast enhancement study also generate non-clinical issues such as additional time and expense for imaging workup. However, a contrast enhancement study might be considered as an imaging workup as it represented high detection rate (4.7%) in our study along with significant management modification in patients with cognitive impairment.

Our study had several limitations. First, decision of contrast media was solely determined by referring physicians which may offer a large potential bias. Though contrast media should be preferred in the patients with a known brain mass, they (30 patients with 30 exams) were excluded from the source population. We additionally tried to reduce potential selection bias using individual matching. Second, this is single-center retrospective study. Detection rates that may be dependent upon disease prevalence may additionally influenced by location and institution. However, this study included a large study population. Third, though we included patients with cognitive impairment and underwent brain MRI, detection rate, the primary outcome of our study was not defined for evaluating causes of cognitive impairment and test positive was defined irrelevantly to the cause of chief complaint. However, we additionally tried to evaluate the proportion of causative lesions among test positives based on its locations. Although whether there was symptom improvement after treatment was also not investigated, we evaluated the proportion of patients who underwent treatment or follow-up imaging for the positive findings. Fourth, although our study included a large population, the frequency of true-positive lesions remained insufficient to evaluate the necessity of contrast enhancement study in imaging workup for cognitive impairment. The evaluation of efficiency in detecting incidental metastases with contrast enhancement study was limited due to the low incidence: 0.50% (6/1,203) with contrast media and 0.09% (2/2,320) without contrast media, respectively, after individual matching. Hence, studies with a larger population with multicenter study are warranted. Fifth, reports without positive results were not reviewed in this study. The use of limited metrics is a limitation in terms of diagnostic accuracy which is usually represented as a combination of sensitivity and specificity. However, we tried to focus on a combination of detection rate and false referral rate, which is more of a term toward diagnosis-related patient outcomes than diagnostic accuracy and usually used in screening test research [13]. Sixth, there was no predefined washout period or blinding between reviewing images with and without contrast media, which is only can be done with prospective study design. Seventh, only single type of contrast media was administered.

## Conclusion

In conclusion, detection rate of brain MRI for lesions only detectable with contrast media in patients with cognitive impairment was not high enough and it is relatively reasonable not to use contrast media in patients with cognitive impairment. Further study is needed to develop a strategy for identifying patients whom would especially benefit with contrast media correlating with the factors such as atypical presentation, known malignancy, or older age.

## Supporting information

S1 Fig Trend in numbers of enhancement in brain MRI for patients with cognitive impairment in recent years (Data based on Table 1). (DOCX)

S1 File (XLSX)

S1 Table Detection rate of MRI with or without contrast enhancement in patients with cognitive impairment by age and sex. (DOCX)

S2 Table Detection rate of MRI with or without contrast enhancement in patients with cognitive impairment by age. (DOCX)
