# Supplementary material for: Detection rate of contrast-enhanced brain magnetic resonance imaging in patients with cognitive impairment
Source: PLoS One. 2023 Aug 7;18(8):e0289638. doi: 10.1371/journal.pone.0289638 (PMC10406288; doi:10.1371/journal.pone.0289638)

**S1 Figure. Trend in numbers of enhancement in brain MRI for patients with cognitive impairment in recent years (Data based on Table 1)**


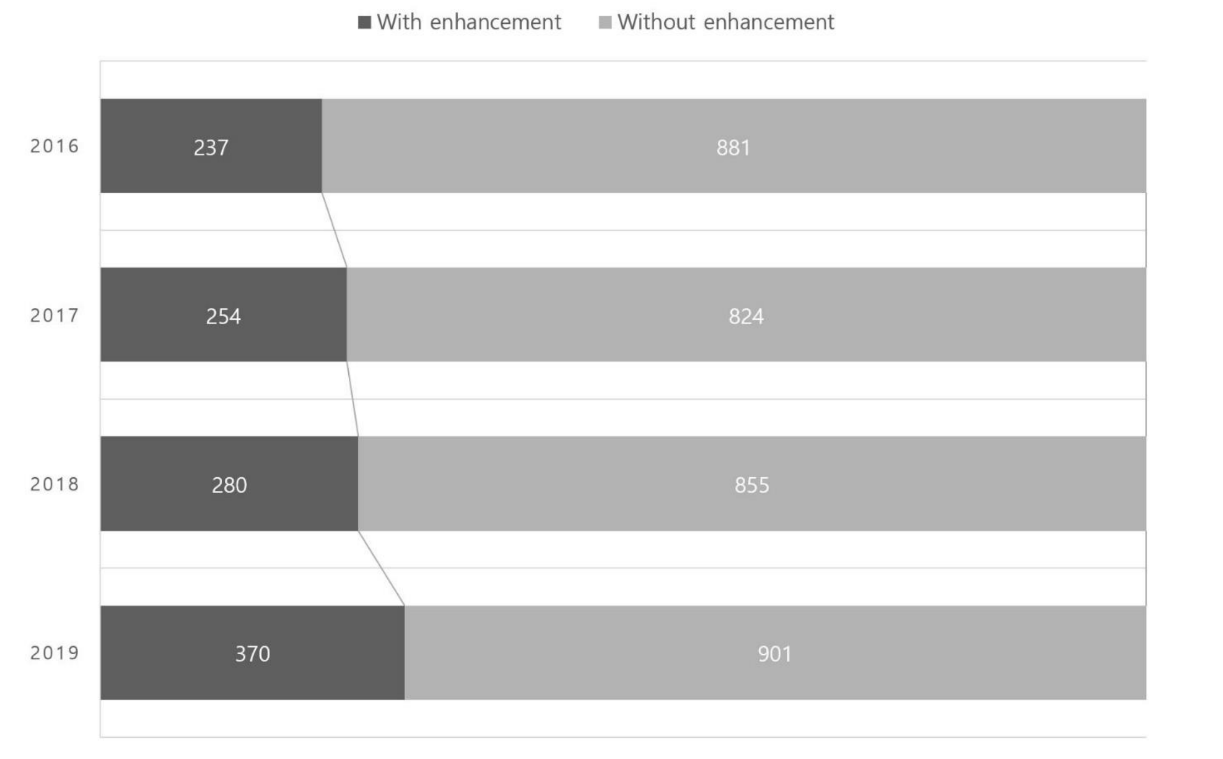

Supplement: S1 Fig — (DOCX) [file pone.0289638.s001.docx]
