# Supplementary material for: Detection rate of contrast-enhanced brain magnetic resonance imaging in patients with cognitive impairment
Source: PLoS One. 2023 Aug 7;18(8):e0289638. doi: 10.1371/journal.pone.0289638 (PMC10406288; doi:10.1371/journal.pone.0289638)
Supplement: S1 Table — (DOCX) [file pone.0289638.s003.docx]

**S1 Table. Detection rate of MRI with or without contrast enhancement in patients with cognitive impairment by age and sex.**

|  |  | **Total (n=4,838)** | | | **Individual matching (n=3,523)** | | |
| --- | --- | --- | --- | --- | --- | --- | --- |
|  |  | **With enhancement**  **(n=1,203) (%)** | **Without enhancement**  **(n=3,635) (%)** | **P value** | **With enhancement**  **(n=1,203) (%)** | **Without enhancement**  **(n=2,320) (%)** | **P value** |
| **Age** | **<70** | 29 (4.7) | 20 (1.4) | <.001 | 29 (4.7) | 19 (1.6) | .001 |
|  | **≥70** | 28 (4.8) | 45 (2.0) | <.001 | 28 (4.8) | 26 (2.2) | .011 |
| **Sex** | **F** | 35 (5.1) | 36 (1.6) | <.001 | 35 (5.1) | 24 (1.8) | <.001 |
|  | **M** | 22 (4.3) | 29 (2.2) | .015 | 22 (4.3) | 21 (2.1) | .034 |
