# Supplementary material for: Detection rate of contrast-enhanced brain magnetic resonance imaging in patients with cognitive impairment
Source: PLoS One. 2023 Aug 7;18(8):e0289638. doi: 10.1371/journal.pone.0289638 (PMC10406288; doi:10.1371/journal.pone.0289638)
Supplement: S2 Table — (DOCX) [file pone.0289638.s004.docx]

**S2 Table. Detection rate of MRI with or without contrast enhancement in patients with cognitive impairment by age**

|  | **Total (n=4,838)** |  |  | **Individual matching (n=3,523)** |  |  |
| --- | --- | --- | --- | --- | --- | --- |
| **Age < 70 years (n=2,017)** | **With enhancement (n=623) (%)** | **Without enhancement (n=1,394) (%)** | ***P* value** | **With enhancement (n=623) (%)** | **Without enhancement (n=1,160) (%)** | ***P* value** |
| **Overall detection rate** | 29 (4.7) | 20 (1.4) | <.001 | 29 (4.7) | 19 (1.6) | <.001 |
| **Subgroup** |  |  |  |  |  |  |
| **Meningioma** | 13 (2.1) | 12 (0.86) | .022 | 13 (2.1) | 11 (0.95) | .067 |
| **Metastasis** | 0 (0) | 1 (0.07) | 1.000 | 0 (0) | 1 (0.09) | - |
| **Glioma** | 1 (0.16) | 2 (0.14) | 1.000 | 1 (0.16) | 2 (0.17) | .953 |
| **Pituitary adenoma** | 1 (0.16) | 0 (0) | .309 | 1 (0.16) | 0 (0) | - |
| **Lymphoma** | 1 (0.16) | 0 (0) | .309 | 1 (0.16) | 0 (0) | - |
| **Neurogenic tumor** | 1 (0.16) | 2 (0.14) | 1.000 | 1 (0.16) | 2 (0.17) | .953 |
| **Others*** | 12 (1.9) | 3 (0.22) | <.001 | 12 (1.9) | 3 (0.26) | <.001 |
| **Treatment or follow-up imaging** | 12 (1.9) | 11 (0.79) | .026 | 12 (1.9) | 10 (0.86) | .074 |
| **Age ≥ 70 years (n=2,821)** | **With enhancement (n=580) (%)** | **Without enhancement (n=2,241) (%)** | ***P* value** | **With enhancement (n=580) (%)** | **Without enhancement (n=1,160) (%)** | ***P* value** |
| **Overall detection rate** | 28 (4.8) | 45 (2.0) | <.001 | 28 (4.8) | 26 (1.2) | .011 |
| **Subgroup** |  |  |  |  |  |  |
| **Meningioma** | 11 (1.9) | 20 (0.89) | .045 | 11 (1.9) | 12 (1.0) | .181 |
| **Metastasis** | 6 (1.0) | 1 (0.04) | <.001 | 6 (1.0) | 1 (0.09) | .028 |
| **Glioma** | 0 (0) | 3 (0.13) | 1.000 | 0 (0) | 0 (0) | - |
| **Pituitary adenoma** | 1 (0.17) | 3 (0.13) | 1.000 | 1 (0.17) | 2 (0.17) | 1.000 |
| **Lymphoma** | 1 (0.17) | 2 (0.09) | .499 | 1 (0.17) | 1 (0.09) | .655 |
| **Neurogenic tumor** | 0 (0) | 2 (0.09) | 1.000 | 0 (0) | 1 (0.09) | - |
| **Others*** | 9 (1.6) | 14 (0.62) | <.001 | 9 (1.6) | 9 (0.78) | <.001 |
| **Treatment or follow-up imaging** | 12 (2.1) | 11 (0.49) | <.001 | 12 (2.1) | 4 (0.34) | .006 |

Note: Logistic regression with generalized estimating equations method was used to compare the detection rate according to the need for contrast enhancement.

MRI = magnetic resonance imaging

* Others: bone tumor, encephalitis, meningitis, vascular malformation, post-ictal change, epidermoid cyst
